# Lotka-Volterra models for the market penetration of renewable energy

**DOI:** 10.1016/j.heliyon.2023.e22704

**Published:** 2023-11-23

**Authors:** Norbert Brunner, Doris Straßegger

**Affiliations:** Institute of Mathematics, Department of Integrative Biology and Biodiversity Research, University of Natural Resources and Life Sciences, Vienna, Austria

**Keywords:** Carbon neutrality, Green electricity, Renewable energy, Logit regression, Lotka-volterra differential equation

## Abstract

We studied trend models for the energy and electricity markets and compared their complexity. Was it associated to the ease of policy interventions to control these markets? Using open data, we applied Lotka-Volterra type models to describe the evolution of the market shares of renewable energy and of electricity from renewable sources and observed that for the latter data more complex models were needed. We then searched for characteristics and anomalies in these models that might indicate immunity to and susceptibility for policy interventions towards reaching carbon neutrality, respectively. However, the evidence linking complexity and receptiveness for policy interventions remained weak.

## 1 Introduction

### 1.1 Context of the paper

There is an international commitment, codified by the Paris Agreement [1], to substantially reduce the use of fossil fuels by 2050 and make the energy sector carbon neutral [2,3]. This means “a balance between anthropogenic emissions by sources and removals by sinks of greenhouse gases” [1]. However, studies based on extrapolations of past trends have shown that the emissions of greenhouse gas continued to rise and there would be no sign that these emissions would peak soon [4,5]. As the extrapolation of past trends means “business as usual”, this indicates a need for a change. Change would be still possible but require considerable efforts [6,7,8]. Technologies that aim at carbon capture for later utilization or storage [9], energy saving technologies, or technologies that replace conventional fuels by renewable ones [10] might be of interest. However, accelerating the decarbonization of the electricity sector would be most important, requiring the expansion of solar photovoltaics (PV), wind power, hydropower, and nuclear power [6]. It follows that the share of renewable energy amongst global energy production needs to rise dramatically. For example [11], projects a scenario, where renewable energy production needs to rise from 12 % of total production in 2020 (69 EJ of 587 EJ, where EJ = exajoule) to 67 % of projected production in 2050 (362 EJ of 543 EJ) to achieve carbon neutrality by 2050.

### 1.2 Goal of the paper

Can the difficulty of the needed change be assessed from past market data? We first inquired about the complexity of the trend lines that describe the temporal evolution of the market shares of renewables (energy or electricity). Our notion of “complexity” was borrowed from the Akaike [12] information criterion: We sought trend lines with few parameters that had an acceptable fit to the data and that were most parsimonious amongst such simple trend lines. We then related the complexity of models to the difficulty of rising the market shares of renewables.

Our hypothesis states that successful policy interventions in the past may have generated data that could be approximated well by complex trends, only. Conversely, we suspected that simple trends with good fits to the data might indicate resistance against attempted policy interventions.

Under this hypothesis, extrapolating information about the complexity into the future, we expect that policies to substantially increase the market shares of renewable energy products might succeed if complex trends were needed for data fitting. However, such policies might fail if simple trends sufficed, unless the trends already forecasted substantially higher shares for renewables. Specifically, we asked under this hypothesis, which policy goals are easier to attain, boosting production of renewable energy or of green electricity.

To ensure comparability of data, we sought for open statistics that respected the UNSD [13] classification of energy products and that in addition differentiated between renewable and non-renewable products. Therefore, we used open data [14] about the production of energy and electricity over three decades. In addition, we used national data of Austria that were more detailed with respect to green energy and green electricity (meaning renewable energy and electricity coming from renewable sources). We then fitted trend lines to these data. For this purpose, we used solutions of the Lotka [15] and Volterra [16] system of differential equations, as they allow to model the possible interactions between the various products.

### 1.3 Relation to other work

Our study focuses on the production of energy and electricity, but not on the resulting eco-balances. Therefore, we do not discuss if in a life-cycle perspective more production of renewable energy may indirectly result in more greenhouse gas emissions (e.g. Ref. [17], for wind energy).

Further [18], pointed out that the greenhouse effect [19,20] would be only one aspect of the environmental degradation due to energy production. Examples for other aspects are acid rain from sulfur dioxide emissions (combustion of coal), changes of landscapes by hydropower, excessive land use from solar farms, potential of serious accidents for nuclear power plants, threats to wildlife from wind turbines, or water pollution from oil and gas (using fracking). Aggregating these environmental impacts into an ecological footprint had surprising conclusions [21]: Using more nuclear and wind energy might reduce the ecological footprint, while the production of geothermal energy and hydropower could rise it.

Our study uses deterministic trend models that aim at identifying structure in smaller datasets. Trend models include general-purpose models, such as linear or quadratic regression, or logistic growth. One of the oldest and still widely used approach for long-term forecasts of energy production is the Hubbert [22] peak theory (Google scholar: 11.400 mentions since 2003), which focuses on depletion. It described the cumulative oil production over time by a logistic growth model (in a different context: [23]). Therefore, fitting a logistic growth curve to cumulated production data (or fitting its derivative to annual production data) would allow to forecast, when oil production will peak and how large the remaining reserves would be [24]. tested this theory and found that in general the theory provided reasonable estimates. However, for different data different trend models should be used. Further, in practical applications there would be high data uncertainties.

Alternative approaches for long-term forecasts use scenario analyses under different assumptions about demographic, political, and economic trends. A highly esteemed example is the Global Energy and Climate model [25] of the International Energy Agency (IEA). This is an international organization that was established in 1974 under the auspices of OECD. Amongst its guiding principles is the goal of “supporting countries in the global effort to attain net zero greenhouse gas emissions in the energy sector by mid-century” [26].

For short-term forecasts, also statistical time-series models are commonly used. Such non-deterministic models range from simple moving averages and exponential smoothing to more complex autoregressive moving average processes and variants (e.g., AR, MA, ARMA, ARIMA). As for an example in energy studies [27], linked the logarithm of per capita energy consumption to economic growth.

However, our goal was not the forecasting of energy production (except for illustrative examples). Rather, we were interested in describing the past evolution of the market shares of renewables. As market-shares are bounded by 0 and 1, linear regression would be unsuitable for long-term forecasts, because the regression line, unless it is constant, would not remain within these bounds. There are different approaches to overcome this difficulty. Logistic regression [28] fits a regression line (or another trend) to the logits, ln (*s*/(1–*s*)) of the market shares, *s* (ln = natural logarithm). A disadvantage of this approach is the consideration of each product in isolation from the others. Bass [29] developed a model that considers the products (their market shares) simultaneously.

Here we consider the Lotka-Volterra (LV) system of differential equations, another general-purpose trend model. Originally, it was used in mathematical biology to derive trends from the interaction of different species [30,31] and in economy to explain business cycles [32]. It was later adopted to analyze the dynamics of markets [33,34,35]. A major advantage of LV type models is the explicit study of the interaction between different products. When different forecasting methods with small data requirements were compared, the LV methodology fared best [36,37]. As for an example in energy studies using the classical autonomous LV system (constant interaction coefficients) [38], concluded from an analysis of the interaction coefficients that promoting the use of low-carbon energy might accelerate fossil fuel consumption. This result was confirmed by life-cycle analyses, such as of nuclear power generation [39].

This paper is based on a particular class of LV models with variable interaction coefficients. It was devised by Ref. [40]. Other than the autonomous LV systems with constant interaction coefficients, these models are capable of modeling exogeneous changes of the competitive environment, such as technological innovation or management initiatives. Technically, the models transform the data into utilities and then they apply nonlinear regression to each of the time series of utilities. Insofar, they generalize the well-known logit model. (It is the special case of one good and the remainder of the market, where the utility coincides with the logit.) Consequently, there are applications in multiple fields, such as tourism [41], transport industry [42], social studies related to intergenerational conflicts [43], grocery business [44], water resources management [45], or epidemiology [46]. The first author used this model in various applications, too, such as in automotive industry [47], food industry [48], or water management [49]. Some more technical comments (Section 2) draw on these experiences, but the applications of the LV model in this paper are new.

In the present context, market shares in units (produced renewables in percent of produced energy in joules) may be more informative than market shares in dollars (sold renewables in percent of energy sales in dollars); see Ref. [35] for this distinction. For, the amount of carbon dioxide emissions from fossil fuels depends on the quantity of the consumed fuels, not on their costs. Further, in view of the long-term contracts typical for the energy sector, we expected that quantities might be more resilient to economic shocks, while prices and thus economic values may be volatile. In this respect, our paper differs from previous LV studies that focused on sales and compared market prices [50,51,52]. For instance, in energy related studies [53] analyzed fossil and renewable energy stock prices and identified a structural break in 2008, when there was a global economic crisis. (Structural breaks in the dynamics occur when the signs of certain interaction coefficients change.) As for another example, according to Ref. [54] policies aiming at reducing greenhouse gas emissions by promoting renewables in the long term might be incompatible with policies aiming at full employment.

Finally, other than the above-mentioned literature, the focus of the paper is on the complexity of the models fitted to past data. We seek to fit simple trend lines to the utilities of past production data and then use LV models to model the market shares.

## 2 Methods

### 2.1 Data

The world energy balances [14] of International Energy Agency (IEA) published timeseries for 49 industrialized and emerging countries and for several regions about 13 flows (e.g., production, electricity output) between 1971 and 2020. The data are available as downloadable Excel file, and IEA permits to display them in publications in graphical form, only, not as tables.

IEA collects its data from its member and associated member countries that response to annual “joint questionnaires”. However, to ensure comparability of countries, international statistics use rather coarse classifications of energy products. For instance, in Ref. [14] renewable energy included hydropower, wind power, solid, liquid or gaseous biofuels (e.g., firewood, biodiesel, biogas), solar power (photovoltaics), geothermal energy and waste (e.g., municipal waste from biological materials). Non-renewables included carbon-based fossils (peat, coal, crude oil, oil shale, natural gas, natural gas liquids, and refinery feedstocks), nuclear fuels, and heat (generated from fossils). The non-renewable sources for electricity were comprised of carbon-based fossils and nuclear fuels.

We did not consider data of [14] for country groups, except for “world”, and we did not consider Argentina, Costa Rica, Egypt, and Ukraine, as data dispayed large blocks of “0”. Thus, we considered Australia, Austria, Belgium, Brazil, Canada, Chile, China, Colombia, Czech Republic, Denmark, Estonia, Finland, France, Germany, Greece, Hungary, Iceland, India, Indonesia, Ireland, Israel, Italy, Japan, Korea, Latvia, Lithuania, Luxembourg, Mexico, Morocco, Netherlands, New Zealand, Norway, Poland, Portugal, Singapore, Slovak Republic, Slovenia, South Africa, Spain, Sweden, Switzerland, Thailand, Turkiye, UK, and USA.

**Fig. 1:**
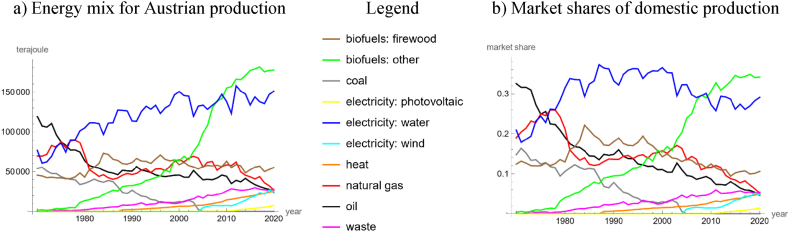
Energy production in Austria, 1970–2020, based on [55]; a) energy mix and b) market shares.

In addition, we considered national data about Austria from Statistics Austria [55], because these data contained more details about renewables (Fig. 1a and b). Owing to Austria's alpine topography and temperate climate, the production of renewable energy has always been important. Almost half of the land surface is covered by forests, whence biofuels are a major source of energy [56]. Another main source is hydropower which was developed by large projects since the 1950s. However, due to increasing awareness for environmental concerns, large projects along river Danube were stopped in 1984, leading in 1996 to the foundation of the Danube-Auen National Park [57]. The harvesting of heat (e.g., solar thermal energy, geothermal heat) is still an emerging source of green energy [58]. Thermal treatment of waste in incineration plants is common and considered as environmentally sustainable [59]. In addition, the production of biogas is emerging [60]; methane from landfills and wastewater treatment is collected, too. With respect to fossil energy, there is a small domestic production of oil and gas [55]. There are reserves of oil shale but owing to environmental concerns there was no production by fracking [61]. Peat was not used for energy production, the production of coal was phased out in 2016, and a law of 1978 (Federal Gazette 676/1978: Atomsperrgesetz) banned nuclear power plants. However, there remained imports of electricity from nuclear power and imports of coal, gas, and oil.

[55] collected data about the yearly domestic production of energy in Austria from 1970 to 2020 (51 years) from a variety of different sources, such as from E-Control (government regulator for electricity and natural gas markets), BM:K (Federal Ministry of Climate Action, Environment, Energy, Mobility, Innovation and Technology), or UBA (Environment Agency Austria). Since 1970, there have been minor changes to the collection methods. Subscribers can access the data online (StatCube of Statistics Austria). We obtained permission to publish the data about domestic energy production as supporting information.

Comparing the data of [55] with those of [14], there were several differences. For example [55], distinguished for biofuels between traditional firewood in the form of logs (biofuels: firewood) and about other biogenic energy products (biofuels: other), which may be produced from wood (e.g., wood pellets) or from other sources (e.g., biodiesel). There were minor differences for coal, gas, and oil. The categories “heat” of [14] and “ambient heat” of [55] were not comparable. Perhaps this was due to the different handling of “heat”. For [14] it meant the production of heat from the combustion of fossil fuels (e.g., process heat), while [55] accounted this for the respective fossil fuels [14]. added geothermal heat to renewable energy, while for [55] this was a component of ambient heat. Further, during the 1980s there were notable differences (up to 25 %) for renewable energy plus waste.

### 2.2 Lotka-volterra model

This outline explains the LV system of differential equations from Ref. [40], equation (1). This system was based on [62]. The solutions, *y*_*i*_, of the following LV system of differential equations aim at describing the evolution of the market shares of products, *i* = 1, 2, …, over time, *t*.

(1) $$\frac{dy_{i}\left(t\right)}{dt}=y_{i}\left(t\right)∙\left(g_{i}\left(t\right)-\sum_{j=1,2,\ldots }g_{j}\left(t\right)∙y_{j}\left(t\right)\right)\;i=1,2,\ldots$$

The interaction coefficients, *g*_*i*_, depend on time and their sign characterizes the market dynamics [37,40,35]: The positive or negative signs of the interaction coefficients *g*_*i*_(*t*) and *g*_*j*_(*t*) in equation (1) indicate that at time, *t*, the respective goods, *i* and *j*, are in a situation of competition (++), predator-prey (+−), or mutualism (−−). Thus, a change of sign of an interaction coefficient indicates a structural break of the market dynamics that could be the result of a crisis, a policy change or a technological innovation.

To fit model (1) to given market data, for each energy product we defined a utility function from its market share, and we identified its trend. Its derivative in turn defined the interaction coefficient, *g*_*i*_ of product *i*. The resulting LV model function, *y*_*i*_, then described the evolution of the market shares over time and could be applied for instance in forecasting. Key steps in the application of the LV model of [40] are the identification of an outside good and the choice of a suitable regression model fitted to the utilities. In general, we identified the outside good (in the sequel, *i* = 0) as the good (group of energy products) with lowest market share. We denoted the observed market share of a good, *i* = 0, 1, …, at time *t*, by *s*_*i*_(*t*). Using it, we then defined time-dependent utilities *u*_*i*_(*t*) for the goods by means of equation (2). Note that the utility of the outside good was nil.

(2) $$u_{i}\left(t\right)=\ln\left(\frac{s_{i}\left(t\right)}{s_{0}\left(t\right)}\right)$$

Next, for each good (*i* = 1, 2, …) we identified a trend underlying the empirical utilities. To this end, we fitted an assumed model function, *v*_*i*_(*t*), to the observed utilities, *u*_*i*_(*t*). The derivatives, *g*_*i*_(*t*) = *v*_*i*_′(*t*), defined the interaction coefficients for the system (1). The solutions of system (1) were the LV model functions, *y*_*i*_(*t*). They were given by equations (3a) and (3b). These solutions then approximated the observed market shares, *s*_*i*_(*t*).

(3a) $$y_{i}\left(t\right)=\frac{\exp\;\left(v_{i}\left(t\right)\right)}{1+\sum_{j=1,2,\ldots }\exp\;\left(v_{j}\left(t\right)\right)}\;i=1,2,\ldots$$

(3b) $$y_{0}\left(t\right)=\frac{1}{1+\sum_{j=1,2,\ldots }\exp\;\left(v_{j}\left(t\right)\right)}=1-\sum_{i=1,2,\ldots }y_{i}\left(t\right)$$

As an advantage, when compared to the conventional approaches using autonomous LV equations, this LV model was not fitted directly to the market shares [63], but it was derived from a fit to utilities (related to the market shares). Insofar, the approach resembles (and generalizes) logit regression, which is an established tool in economic studies: In a market with two products, such as 1 = renewable energy and 0 = outside good = non-renewable energy, the utility of good 1 coincides with the logit of the market share (where *s*_0_ = 1–*s*_1_). Further, there are no issues about model curves predicting unreasonable market shares, i.e., less than 0 % or more than 100 %. (By contrast, consider the best fitting interaction parameters for an autonomous system of LV equations, meaning minimal sum of squared errors, added over all model curves. Using these parameters and a slightly perturbated initial value may result in a trajectory that generates unreasonable market shares.)

We preferred simple trend lines (with few parameters) to model the utilities, such as linear, quadratic, or cubic polynomials with two to four parameters, (4a), rational trends with three parameters, (4b), or trigonometric trends with four parameters (4c). In addition, we considered bilinear trends with four parameters, (4d), trigo-linear trends with up to five parameters, (4e), and trigo-rational trends with six parameters, (4f).

(4a) $$v\left(t\right)=\sum_{m=0}^{n}a_{m}∙t^{m}$$

(4b) $$v\left(t\right)=a∙\frac{1+b∙t}{1+c∙t}$$

(4c) v(t)=a+b∙cos(c∙t+d)

(4d) v(t)=max{a+b∙t,c+d∙t}

(4e) $$v\left(t\right)=a+b∙t+c∙\cos\left(\frac{2∙\pi }{e}∙t\right)+d∙\sin\left(\frac{2∙\pi }{e}∙t\right)$$

(4f) $$v\left(t\right)=a∙\frac{1+b∙t}{1+c∙t}+d∙\cos\;\left(e∙t+f\right)$$

Our models fitted the same type of trend lines to the utilities of all goods (groups of energy products). Polynomial trends were suggested by [[44]; 43]. In the special case of linear trends for the utilities, the LV model (1) was an autonomous system with constant coefficients. We used the rational trend to improve the linear trend while avoiding surprising changes in the general direction of the trend. We did not extend the rational trend beyond its pole (at −1/*c*); we dismissed the trend if there were data points to the left and to the right of the pole. The trigonometric trend was the simplest case of a Fourier series, which was suggested for energy studies [53]. Thereby, we distinguished between Fourier series, where the period length was given in advance (as for classical Fourier series) and Fourier series, where the period length was optimized along with the other parameters (as for the trigonometric trend). Further, we combined trends. For example, trigo-linear or trigo-rational were sums of a linear or a rational and a trigonometric tend. The rationale was the modeling of deviations of a linear or rational trend due to a hypothetical “business cycle”. A bilinear trend was a continuous piecewise linear function, which glued together two linear trends.

### 2.3 Data fitting and goodness of fit

Following [64], we used nonlinear regression (method of least squares) to fit model curves, *v*_*i*_(*t*), to the observed utilities, *u*_*i*_(*t*). We thereby sought parameters for the model curves that for each good, *i*, minimized the sum of squared errors, *SSE*_*i*_ of equation (5), whereby *t*_*k*_ (for *k* = 1, 2, …, *K*) were the time coordinates of the considered data points.

(5) $${SSE}_{i}=\sum_{k=1}^{K}{\left(v_{i}\left(t_{k}\right)-u_{i}\left(t_{k}\right)\right)}^{2}\;i=1,2,\ldots$$

Unless stated otherwise, we used Mathematica 13.3 [65] for our computations and plots. (The data were imported from spreadsheets: Microsoft Office Excel.) For nonlinear regression we used its command “NonlinearModelFit”, selecting different optimization methods. (If different methods generated different outcomes, we reported the results with the simplest appearance.) Our preferred method was a random search: It generated parameter-values at random (we required 1000 search points). Starting from them, it used common local optimization methods, such as Newton-style methods. Amongst the so obtained local optima, the best one was chosen as the solution. We improved it further, using another (slower) optimization method (interior point method [66]). To specify this optimization method, a nested option was used:

Method→{"NMinimize", Method→{"RandomSearch", "SearchPoints"→1000, “PostProcess”→"InteriorPoint"}}

We used R-squared of equation (6) to report the goodness of fit of the regression line that modeled the trends underlying the utilities. R-squared relates *SSE* for the fitted model to *SSE* for the trivial constant function model.

(6) $$R_{i}^{2}=1-\frac{{SSE}_{i}}{\sum_{k=1}^{K}{\left(u_{i}\left(t_{k}\right)-\text{mean}\left(u_{i}\left(t_{1}\right),u_{i}\left(t_{2}\right),\ldots \right)\right)}^{2}}\;i=1,2\text{,}\ldots$$

To report the goodness of the fit of the resulting LV model functions, *y*_*i*_, to the observed market shares, *s*_*i*_, we used the precision rate of equation (7); it is 1 minus the mean absolute relative error. The precision rate was suggested in Ref. [36].

(7) $${\mathrm{PR}}_{i}=1-\frac{1}{K}∙\sum_{k=1}^{K}\frac{\left|s_{i}\left(t_{k}\right)-y_{i}\left(t_{k}\right)\right|}{s_{i}\left(t_{k}\right)}\;i=0,1,2\text{,}\ldots$$

We reported these measures, as they could be compared across different data. We did not report R-squared for the market shares, as the LV model curves for the market shares were not necessarily the best-fit least squares approximations to the data.

For model selection, the Akaike [12] information criterion, *AIC*, has been recommended [67]. Equation (8) defines *AIC* from *SSE* [68]. Thereby, *K* is the count of data and *P* is the number of optimized parameters (parameters of the model equation plus one, as *SSE* is optimized, too). If several models for the same data are compared, and a priori there is no reason to exclude a model, then the model with the least *AIC* is the most parsimonious model that shall be selected.

(8) $$AIC=K∙\ln\left(\frac{SSE}{K}\right)+2∙P$$

Best-fit parameters of the regression lines were maximum-likelihood estimators, if the fit errors were independent and identically normally distributed random variables, i.e., “white noise” [69]. In this case the definition of *AIC* by equation (8) was justified, as the maximum likelihood could be computed from *SSE*. We therefore tested the “white noise” assumption using the Cramér & von Mises test for normal distributions and the Box & Pierce [70] test for significant autocorrelations. A failure of these tests could indicate a misspecification of the model. (Moreover, the confidence intervals could underestimate the variability of the best-fit parameters [71], whereas the fulfillment of the “white noise” hypothesis may not always exclude excessively large confidence intervals. As for an alternative approach [23], supplemented trend models by a stochastic process to model the fit errors.).

Summarizing, we deemed the fitted model as acceptable, if the following four conditions were satisfied: For the fit errors (differences between trends and logits), the P-value of the Cramér & von Mises test for normality was below 0.01. The P-value of the Box & Pierce test for autocorrelations was below 0.01. For the trend line, R-squared (*R*^2^) for the fit to the logits was above 0.8. For the LV model function describing the market shares, the precision rate (*PR*) was above 0.8. These conditions for a good fit were not very restrictive for a single trend line. However, in the case of the logit models, we fitted trend lines to many time-series, and in the case of the more general LV models, each model involved several time-series to which trend lines were fitted. In both cases, we wanted to avoid spurious refutations of models. In other cases, we defined significance by a threshold of 0.05 for the P-value.

Thus, our approach differs from common LV model studies insofar, as we put more emphasis on simple models and more emphasis on the goodness of fit. In general, if one of the above-mentioned four conditions for the goodness of fit failed, we searched for a different model based on another class of utility functions. However, a low R-squared did not always result in a refutation, as the trend line might be close to constant. Further, in exceptional cases we removed certain datapoints as outliers. We identified them from a visual inspection of the data. To verify this observation, we used the single deletion variances provided by the “NonlinearModelFit” command. The datapoint with the smallest single deletion variance was insofar strange, as its removal would improve the fit. We removed it if there were 9 or more datapoints. (This was suggested by a sign test: If *n* ≥ 8 and the minimum 0 is compared with the median of the range {0, 1, …, *n*–1}, then *p* < 0.01 for the P-value of a sign test.)

## 3 Results

For the international data [14], we used the time series from 1990 to 2019 (30 years) for energy and the time series from 1992 to 2019 (28 years) for electricity, because earlier data were not available for several countries. We did not use the 2020 data for the calibration of the models and instead compared the forecasts of the models with the actual market shares.

For Austrian data [55], we disregarded data prior to 1980. Earlier data might distort the trends by obvious outliers, as several crises shattered the energy market. These crises resulted in substantial drops in global oil production and rising energy prices (1973 oil crisis, when OPEC proclaimed an oil embargo, 1979 revolution in Iran, 1980 Iraq-Iran war). As forecasting served only illustrative purposes and as the used models described up to ten time-series over as few as only ten years, for [55] we did not dispense with the 2020 data.

### 3.1 Logit models

We started with a study of the market shares of renewable energy and of electricity from renewable sources amongst the total worldwide production of energy. In this context, the LV models of the type [40] reduced to fitting logit models to these data, because we compared merely two products, renewables and non-renewables (the outside good).

#### 3.1.1 Energy

We used the data from the [14] world energy balances and considered trends for 1990 to 2019 (30 years) and for 2005 to 2019 (15 years). To this end, we fitted simple trends (linear, quadratic, cubic, rational, and trigonometric) to the logits of the market shares (utilities) of renewable energy (including waste). To retrieve the data for energy from Ref. [14], in this Excel file we selected the country (e.g., “world”), the flow “production (PJ)”, and the products of interest, e.g., “renewables and waste” and “total”. The market shares of renewable energy where the outcomes for renewables divided by the totals. Amounts of energy were reported in petajoule (PJ), whereby 1 J (joule) = 1 Ws (watt-second) is the energy unit of ISU (International System of Units) and 1 PJ = 1000 TJ (terajoule) = 10^15^ J. Further, 1 EJ (exajoule) = 1000 PJ.

**Fig. 2:**
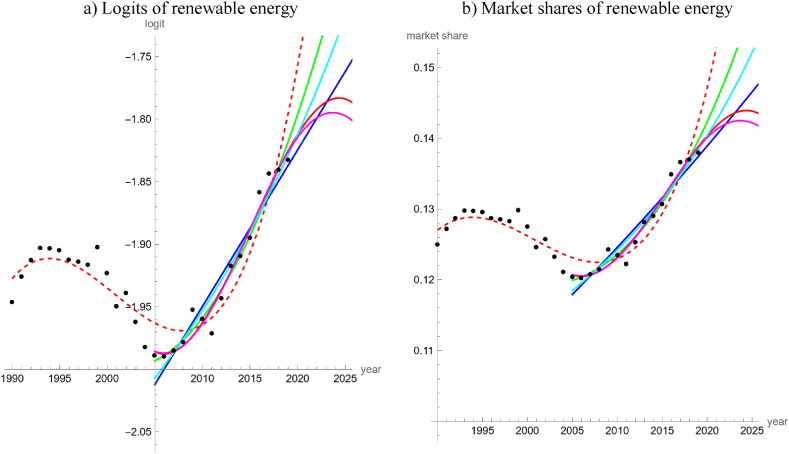
Logits (a) and market shares (b) of renewable energy amongst global production (black dots, based on [14]) and models with an acceptable fit: cubic trend fitted to the 1990–2019 data as dashed red line and five trends fitted to the 2005–2019 data as blue (linear), green (quadratic), red (cubic), cyan (rational) and magenta (trigonometric) lines. Models without an acceptable fit were not displayed. (For interpretation of the references to color in this figure legend, the reader is referred to the Web version of this article.)

For the 30-years data about worldwide energy production, only one of the simple models achieved an acceptable fit to the data, the model defined from the cubic trend line. As the logit data (Fig. 2a) formed a horizontal “S” (peak in 1993, dip in 2001), the shapes of the four other types of simple trend lines were barely compatible with this shape. Computationally, this was reflected by low values of R-squared (while *R*^2^ = 0.86 for the cubic trend). For the 15-years data all simple trends achieved acceptable fits, whereby the trigonometric trend was most parsimonious amongst the five trend lines, followed by the cubic trend. Further, the trigonometric and the cubic trends achieved good fits both to the logits (*R*^2^ > 0.97) and (Fig. 2b) to the market shares (*PR* > 0.99).

For illustrative purposes we extrapolated the trend lines for the worldwide energy production. However, the forecasts did depend both on the models and on the used data. First, we compared the forecasted market shares of renewables in 2020 with the actual market share of 14.8 %. The forecast by the cubic trend based on the 30-year data was most accurate, predicting 14.7 %, followed by forecasted 14.2 % by the quadratic trend for the 15-years data, 14 % by the trigonometric, cubic and rational trends, and 13.9 % by the linear trend. Next, we asked if renewables could reach 90 % market share by 2050 if past trends would continue. The outcome was ambiguous. Again, the cubic trend based on the 30-year data was most optimistic. It forecasted that energy from renewable sources would reach 90 % market share in 2047. The rational, quadratic, and linear trends based on the 15-years data forecasted 90 % market-share, too, but later (in 2060, 2083 and 2341). However, the two most parsimonious trends based on the 15-years data, cubic and the trigonometric, predicted that 90 % market shares would be unattainable for renewables and instead their market shares would start to decline soon (<2025).

We supplemented this analysis by studies of the forty-five countries mentioned in Section 2. Of them, five countries were not considered, as they produced already 100 % renewable energy (Iceland, Latvia, Luxembourg, Portugal, and Singapore). The outcomes suggest that simple models may suffice for the modeling of past energy production. For 57 of the considered 80 datasets (15-years and 30-years data for 40 countries) at least one simple trend achieved an acceptable fit (more information in the Supporting Information). The trigonometric trend was most parsimonious for 25 of these 57 datasets, the cubic trend for 18, the rational one for 8, the linear for 4, and the quadratic for 2. Considering countries, for 37 of 40 countries at least one of the simple models did achieve an acceptable fit to at least one of the 15-years data or the 30-years data. (The exceptions were Morocco, Sweden, and Switzerland.) Thereby, for 27 countries, at least one of the simple trends was acceptable for the 30-year data from 1990 to 2019. For eight countries all five trends fitted well. For 30 countries at least one simple model was acceptable for the 15-year data from 2005 to 2019. For three of them (Austria, Brazil, New Zealand), all five trends achieved an acceptable fit. Austria was the sole country, where all five simple trends fitted well to both the 15-years and to the 30-years data.

For most countries the forecasts about achieving 90 % renewable energy production by 2050 were rather ambiguous or pessimist. Focusing on the most parsimonious trends, they forecasted 90 % market share by 2050 for ten countries and refuted this forecast for 17 countries, when using the 30-years data. Using the 15-years data, the numbers were seven and 23, respectively. Finally, we considered the countries, where both the 15-years and the 30-years data had simple trend lines with acceptable fit. For them, we checked the forecasts for 2050 from the most parsimonious trends. For two countries (Austria, Netherlands) both trends (15 and 30-years data) confirmed that 90 % renewable energy were achievable, while for eight countries both trends rejected this.

#### 3.1.2 Electricity

Next, we used [14] for a study of the market shares of green electricity amongst the total worldwide production of energy. We considered a long time series from 1992 to 2019 (28 years) and a short one from 2005 to 2019 (15 years). To retrieve the data about electricity production from Ref. [14], in this Excel file we selected the country (e.g., “world”), the flow “electricity output (GWh)”, and the products, e.g., “renewable sources” and “total”. Electricity production was reported in gigawatt-hours (GWh), whereby 1 GWh = 10^6^ kWh (kilowatt-hours) = 3.6 TJ and 1 TJ = 0.28 GWh. There was a small difference between total electricity output and the sum of the components. (There was no such difference for energy.) In this subsection, we added this remainder to non-renewable electricity and in Subsection 3.2.3 we defined it as the outside good.

For the 28-years data, the logits and the market shares of electricity from renewable sources basically had the shape of an “U” (vertex in 2003). Three trend lines had an acceptable fit, whereby cubic was most parsimonious, followed by quadratic and trigonometric. For all accepted models, *R*^2^ > 0.955 and *PR* > 0.98. For the 15-years data, all five simple trend-lines achieved acceptable fits with *R*^2^ > 0.99 and *PR* > 0.99 (except for the linear trend). Cubic and trigonometric trends were most parsimonious.

**Fig. 3:**
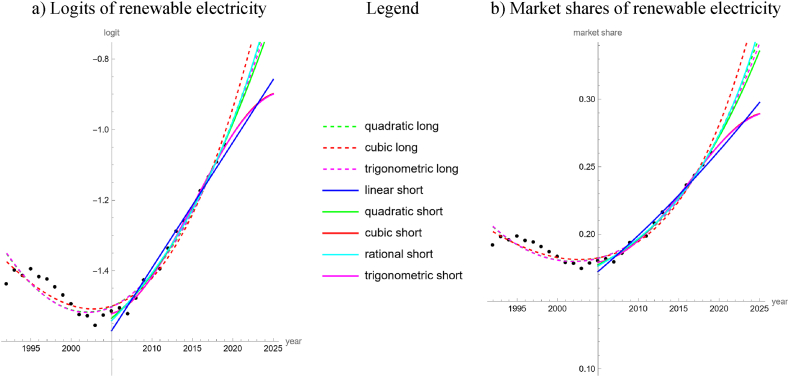
Logits (a) and market shares (b) of renewable electricity amongst global production (black dots, based on [14]). Three logit models were fitted to the 1992–2019 data (“long”) and five to the 2005–2019 data (“short”); models without acceptable fit were not displayed.

Other than for energy, the trend lines for electricity were close together (partially overlapping and therefore not always visible in Fig. 3a and b), especially during 2005–2019. In 2020, green electricity had a market share of 28.0 %. The forecasts from the trend lines for 2020 ranged from 26.2 % to 28.1 %; median 27.25 %. The trend lines fitted to the shorter timeseries predicted rather lower market shares (26.2 % trigonometric, 26.6 % linear and quadratic, 27.2 cubic, 27.4 rational), while the trend lines fitted to the longer time series were more optimist (27.3 trigonometric, 27.4 cubic, 28.1 quadratic). For long-term forecasts the outcomes were ambiguous. The three trend lines with an acceptable fit to the 28-years data forecasted that between 2041 and 2056 green electricity would reach a market share of 90 %. Further, the quadratic, rational and linear trends fitted to the 15-years data forecasted that this market share would be reached between 2037 and 2053. However, the two most parsimonious models fitted to the 15-years data anticipated that 90 % market share would be unattainable.

We did a similar study for the 45 countries mentioned in Section 2. We considered all of them, as there was no country with 100 % green electricity. For 45 of the considered 90 datasets (15-years and 28-years data for 45 countries), at least one simple model achieved an acceptable fit (more details in the Supporting Information). Thereby, the trigonometric model was most parsimonious for 16 of 45 datasets, followed by the cubic one for 12, the linear for 8, the rational for 5, and the quadratic for 4. For Canada, all models achieved an acceptable fit to the 15-years data and for Germany and Iceland, all models achieved an acceptable fit to the 28-years data. However, for 15 of the 45 considered countries, none of the five simple models achieved an acceptable fit to any of the 15-years or 28-years-data. In the long run, considering the countries, where both the 15-years and the 28-years data had simple trend lines with acceptable fit, and asking the respective most parsimonious trends, if 90 % market share for green electricity could be achieved by 2050, then for three countries (Greece, Korea, Thailand) both trends confirmed this, while for four countries (Belgium, Canada, Czech Republic, Ireland) both trends rejected this.

### 3.2 Lotka-volterra models

To obtain a better understanding of the market dynamics, we used LV models to analyze the market interactions of different groups of energy and electricity. We considered global production and the case of Austria. For the latter data we could also investigate the interactions between different types of renewables.

#### 3.2.1 Energy: worldwide

**Fig. 4:**
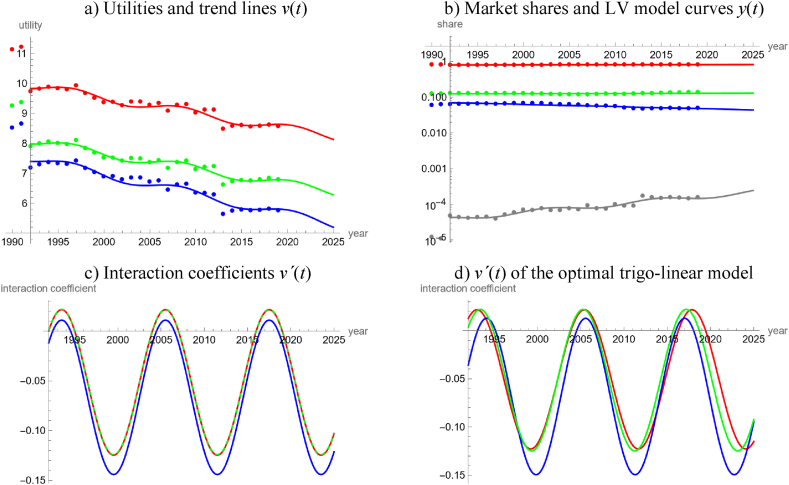
LV model for the global production of four groups of energy products during 1992–2019, based on data from Ref. [14] and model parameters in Table 1. Data (utilities and market shares) are displayed as dots and model curves as lines. Colors identify product groups: red (carbon-based fossils), blue (nuclear), green (renewables and waste) and gray (heat as outside good). Fig. 4a displays the utilities. Fig. 4b is a logarithmic plot of the market shares. In Fig. 4c of the interaction coefficients, the red and green lines overlap. Fig. 4d plots the interaction coefficients for the trigo-linear model of step 2 that optimizes five parameters for each good (energy product group). (For interpretation of the references to color in this figure legend, the reader is referred to the Web version of this article.)

For the worldwide energy production, we used the data [14]. We arrived in the following three steps at the model outlined in Table 1 and Fig. 4. Thereby we considered four groups of energy products: “fossils” (carbon-based fossil fuels), “nuclear”, “green” (renewables and waste), and “heat”, which we defined as outside good, because its market share was small.

**Table 1:** LV model for worldwide energy production.

product group	$v\left(t\right)=a+b∙t+c∙\sin\left(\frac{2∙\pi }{12}∙t\right)-c∙\cos\left(\frac{2∙\pi }{12}∙t\right)$			White noise?		Good fit?	
	*a*	*b*	*c*	*P*_*norm*_	*P*_*auto*_	*R*^2^	*PR*
Fossils	112.5	−0.05	0.099	0.35	0.76	0.940	0.995
Nuclear	140.1	−0.07	0.105	0.15	0.09	0.947	0.950
Green	109.9	−0.05	0.099	0.24	0.43	0.947	0.967
Heat	NA (outside good)						0.912

**Note:** Using the data [14] as described in the text, the table displays the best fit parameters (rounded to the last shown decimal) of trigo-linear trend lines and informs about the goodness of fit.

In a first step, using the 30-years data 1990 to 2019, the fits to the utilities were not satisfactory for simpler models, whence we finally fitted trigo-linear functions, equation (4e), using five parameters each (*a*, *b*, *c*, *d*, *e*) for carbon-based fossils, nuclear and green. The trend lines were composed of linear trends and slight cyclic perturbations. Simpler trend lines without cycles violated the “white noise” hypothesis for certain product groups. However, as is displayed in Fig. 4a, the 1990 and 1991 data appeared like outliers, when compared to the other data, whence we removed them. The reason were the comparably low market shares of heat in 1990 and 1991 (Fig. 4b). These outliers did not matter for the logit analysis of Fig. 2, as the market share of heat was so low.

In the second step, we fitted trigo-linear functions to the utilities for the 28 years 1992–2019 (we had removed the outliers). The optimal periods (*e*) were close to 12 years, namely 12.42, 11.45, and 11.91 years for the utilities of carbon-based fossils, nuclear energy, and renewables, respectively. This suggests that the model might be simplified by using a common period for all products. Further, the ratios *d*/*c* of the parameters in (10) were close to −1, which suggests that the trends moved synchronous. The plot of the interaction coefficients (compare Fig. 4c and d) supports these suggestions.

Finally, in the third step we used this information to simplify the model and arrive at a simple model with three optimized parameters (*a*, *b*, *c*) for each energy product: We defined a common period of *e* = 12 years (for all goods) and defined *d* = –*c*. We repeated the data-fitting for this type of trend lines (Fourier-linear models). Although this assumption reduced the accuracy slightly, because the assumed period was not optimal, for all product groups this Fourier-linear model was more parsimonious than the model with an optimized period (smaller *AIC* owing to fewer optimized parameters). Table 1 and Fig. 4 inform about the outcome of this step 3: There were no problems with the “white noise” hypothesis (for all products and tests, *P* > 0.09) and the fits were good (for all product groups, *R*^2^ > 0.94 and *PR* > 0.95; for the outside good *PR* > 0.91). Thus, using in total nine parameters we could adequately describe four time series over 28 years, each. The selected parameters *e* and *d* were also approximately optimal. (Letting the common period, *e*, vary between 11 and 13 years, and the ratios *d*/*c* between −0.5 and −1.5, using increments of 0.1 in both cases, then the maximum of the three *AIC*-differences between the trigo-rational model that optimized five parameters for each product and the Fourier-linear model that optimized three parameters for each product, was minimal for the period of 12 years and the ratio of −1.)

We used the interaction coefficients for an analysis of the market dynamics. As follows from Fig. 4c, for most of the time all forms of energy production evolved in mutualism (negative interaction coefficients, *g*_*i*_), interrupted by short periods of competition (positive interaction coefficients), and even shorter intermediate periods of predator-prey dynamics (nuclear energy as prey with negative interaction coefficient, renewable and non-renewable carbon-based fuels as competing predators with positive interaction coefficients). Further, considering the linear trends, the slope (*b*) for nuclear fuel was significantly lower than the slopes for carbon-based fossils and green energy (non-overlapping 95 % confidence intervals for the best-fit parameters).

Therefore, extrapolating the past trends for the LV model, nuclear fuels may lose market shares, while renewable and non-renewable carbon-based fuels may slowly gain market shares. (However, forecasted market shares of renewable energy will not reach 90 % by 2050.) The market shares for 2020 were forecasted 82.2 % vs. actual 80.3 % for carbon-based fossils, 4.8 % vs. 4.9 % for nuclear, 12.9 % vs. 14.8 % for renewables, and less than 0.1 % in both cases for heat.

#### 3.2.2 Energy: Austria

**Fig. 5:**
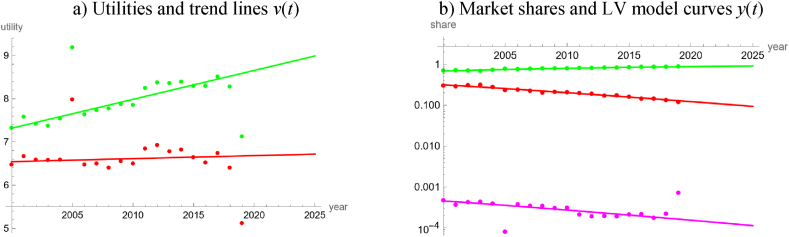
LV model for the energy production of Austria during 2000–2019 in terms of utilities (Fig. 5a) and market shares (5b), based on the data from Ref. [14], disregarding 2005 and 2019. Trends defined in Table 2. Data (utilities and market shares) are displayed as dots and model curves as lines. Colors identify product groups: red (carbon-based fossils), green (renewables and waste), and magenta (heat as outside good). Figure b is a logarithmic plot. (For interpretation of the references to color in this figure legend, the reader is referred to the Web version of this article.)

We repeated this analysis for Austria, using [14] and a short 20-years timeseries starting in 2000. We considered the product groups carbon-based fossils, green energy (renewables and waste), and heat; there was no nuclear energy. As above, we defined heat as the outside good. However, owing to the short time-series, we already succeeded, when we fitted linear trends to the utilities. (Thus, three time-series were adequately described by a model with four parameters.) The outcome is summarized in Table 2 and Fig. 5.

**Table 2:** LV model for domestic energy production of Austria.

Product group	v(t)=a+b∙t		White noise?		Good fit?	
	a	b	P_norm_	P_auto_	*R*^2^	PR
Fossils	−7.3	0.007	0.940	0.057	0.065	0.943
Green	−126.2	0.067	0.077	0.161	0.885	0.985
Heat	NA (outside good)					0.867

**Note:** Using the data [14] for 2000–2019, disregarding 2005 and 2019, the table displays the best fit parameters (rounded to the last shown decimal) and informs about the goodness of fit.

We arrived at this model in two steps. First, we identified two outliers: Fitting a linear model to the utilities for carbon-based fossils, the single deletion variance was lowest for 2019, and fitting a linear trend to the data for green energy, the single deletion variance was lowest for 2005. Therefore, we removed the 2005 and 2019 data as outliers. The common reason for both outliers were the market shares for heat that (for unknown reasons) were exceptionally low in 2005 and exceptionally high in 2019 (Fig. 5b). Consequently, the utilities of fossil and green energy (defined from these market shares) deviated distinctly from the general trend (Fig. 5a).

Second, we fitted linear trends to the remaining data. As the trend lines for the utilities were linear and increasing (Fig. 5a), the LV model (1) had constant and positive interaction coefficients, indicating a competition between production of green and fossil energy. Thereby, the slope *b* of the trend line for fossils did not differ significantly from 0, while the slope for green energy was significantly positive (95 % confidence limits). The white noise hypothesis for the fit errors was not refuted and fits of the model curves were acceptable, except for the small *R*^2^ for fossils. As the trend line for fossils was close to constant, we did not refute the model. The market shares (Fig. 5b) for domestic energy production in 2020 were forecasted 12.2 % vs. actual 10.0 % for carbon-based fossils, 87.8 % vs. 89.9 % for renewables, and less than 0.1 % in both cases for heat.

Using the data of [55] we could study the long-term trends in more detail. We confined the analysis to the 41 data from 1980 to 2020, as these data were no longer affected by the energy crises of the 1970s. For our model, we formed four groups of energy products of assumed similar characteristics: carbon-based fossils (coal, gas, oil), traditional renewable energy (hydropower, firewood), emerging renewable energy (other solid and liquid biofuels, heat, wind power, PV), and waste. We defined waste as outside good. This avoided data uncertainties, as municipal and industrial waste (used to produce electricity and heat) could originate both from renewable resources (e.g., wood residues) and from fossil resources (e.g., plastic bags).

**Fig. 6:**
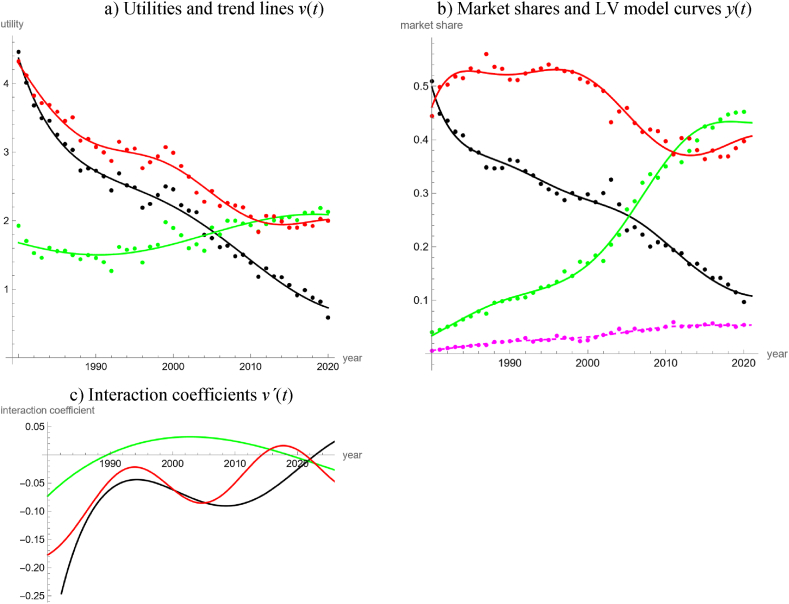
LV model for the domestic production of four groups of energy products during 1980–2020, based on data [55] and model parameters in Table 3, displaying the utilities (6a), market shares (5b), and interactiin coefficients (5c). Data as dots, model curves as lines, colors: black for fossil energy, red for traditional renewable energy, green for emerging renewable energy, and magenta (dashed line in Figure b) for waste as outside good. (For interpretation of the references to color in this figure legend, the reader is referred to the Web version of this article.)

As simple trends were not suitable for all time-series, we finally fitted trigo-rational functions, equation (4f), to the utilities. Table 3 and Fig. 6a, b, and 6c, summarize the results. There was no problem with the “white noise” hypothesis and the fit was good. Only R-squared for emerging renewables was low, as the utility was close to a constant. The added cycles for fossils, emerging, and traditional renewables had periods of 42, 98, and 25 years, respectively, whence we did not use a common period length. Further, we used “100” in the denominator of the rational term to ensure that parameters had comparable sizes, easing the optimization.

**Table 3:** LV model for long-term data about domestic energy production of Austria.

Product group	$v\left(t\right)=a∙\frac{1+b∙t}{100+c∙t}+d∙\cos\;\left(e∙t+f\right)$						White Noise?		Good fit	
	a	b	c	d	e	f	P_norm_	P_auto_	*R*^2^	PR
Fossils	0.8857	−0.0022	−0.0508	−0.3847	0.1480	2.5181	0.24	0.87	0.99	0.96
Emerging	−0.0025	0.7628	−0.0512	−0.9880	0.0641	−0.9400	0.34	0.83	0.80	0.96
Traditional	−0.0059	0.5876	−0.0513	−0.1718	0.2563	−0.2544	0.47	0.61	0.98	0.98
Waste	NA (outside good)									0.93

**Note:** Using data [55] about energy production during 1980–2020, the table informs about the best fit parameters (rounded to the last shown decimal) of trigo-linear trend lines and the goodness of fit.

As follows from Fig. 6c, from 1980 to 1989, all forms of domestic energy production evolved in mutualism. For fossils and traditional renewables, mutualism continued till 2014, whereas in relation to emerging renewables there was a predator-prey situation with the emerging renewables as the predator. Between 2014 and 2018, emerging renewables and traditional renewables were in a state of competition, and both were predators on fossil energy. Finally, by the mid of 2018 traditional renewables became the sole predator, whereas fossils and emerging renewables evolved in mutualism. Further, the model forecasted that starting around 2022 fossils would become the sole predator for more than a decade. (This scenario, and the resulting gain in market shares for fossils, cannot be completely ruled out, as there are currently unused reserves of shale gas.) We conclude for Austria, that in the past the market penetration of renewables was driven by emerging renewables. During 1989–2014 they were the sole predator, with a peak of their interaction coefficient by the end of 2002, but since then they seem to have lost their competitive advantage.

The slope of the trend line of the utility for emerging renewables was rather flat and after 2018, the slope of the trend line of the utility of traditional renewables was flat, too. Hence the competition and the predator prey situations were not clearly pronounced. Consequently, there was some variability for these results: If we fitted the model to the data from 2005 to 2020, then the change of sign of the interaction coefficient for traditional renewables occurred later in 2015. Further, if both renewable groups were combined into one, then fossil energy and the larger group of renewables evolved in mutualism since 1980. Moreover, the parameter confidence intervals were excessively large.

**Fig. 7:**
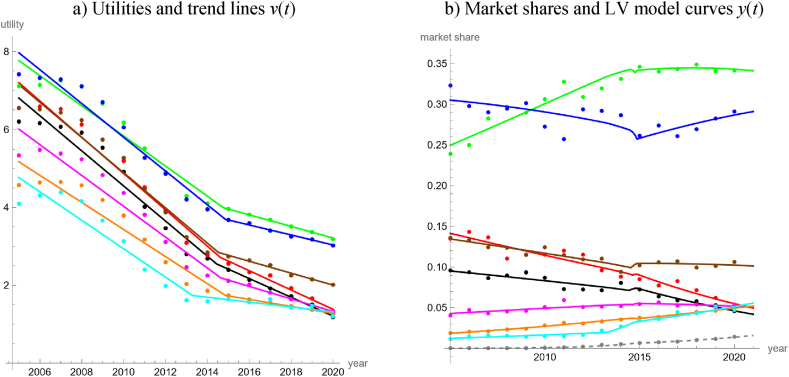
LV model for the domestic production of energy products, 2005–2020, in terms of utilities (7a) and market shares (7b), based on [55] and parameters in Table 4; colors as in the legend for Fig. 1, with the outside good in gray with dashed lines. (For interpretation of the references to color in this figure legend, the reader is referred to the Web version of this article.)

Finally, to fully utilize the detailed information of [55], we considered the time span from 2005 to 2020, when all renewable energy products mentioned in Fig. 1 had been established. We identified photovoltaics plus coal (coal was already marginal) as outside good, as its market share was lowest. In view of the short time series (16 datapoints), we searched for a good-fitting simple model with few parameters. We used the bilinear model with four parameters, as for each of the eight time-series (of inside goods) it was more parsimonious than the LV model based on quadratic trends with three parameters per time-series (smaller *AIC* despite the additional parameter). It delivered an adequate description of nine time-series, using 32 parameters. Table 4 and Fig. 7a and b, summarize the results. The competitive pattern was mutualism between each two energy products (except for the outside good). The bilinear model extrapolated this pattern into the future. Thus, under this model no renaissance of fossil energy production was to be expected.

**Table 4:** LV model for short-term data about domestic energy production of Austria.

Energy product	v(t)=max{a+b∙t,c+d∙t}				Vertex	White Noise?		Good fit	
	a	b	c	d		P_norm_	P_auto_	*R*^2^	PR
Wind power	739.41	−0.3664	133.61	−0.0655	2013.24	0.657	0.608	0.946	0.913
Crude oil	916.71	−0.4538	489.45	−0.2417	2014.36	0.143	0.384	0.976	0.956
Natural gas	951.37	−0.4709	518.22	−0.2559	2014.40	0.848	0.444	0.980	0.940
Firewood	921.44	−0.4560	319.24	−0.1571	2014.42	0.336	0.383	0.976	0.972
Other biofuels	787.53	−0.3889	329.70	−0.1616	2014.56	0.283	0.392	0.964	0.975
Waste	800.67	−0.3963	322.17	−0.1588	2014.66	0.377	0.461	0.969	0.959
Heat	707.55	−0.3503	203.88	−0.1003	2014.71	0.251	0.358	0.960	0.977
Hydropower	875.65	−0.4328	285.09	−0.1396	2014.77	0.230	0.462	0.977	0.966
PV + coal	NA (outside good)								0.812

**Note:** Using data from Ref. [55] about energy production during 2005–2020, the table displays the best fit parameters (rounded to the last shown decimal) of bilinear trends and informs about the goodness of fit. The products are ordered by the vertices (jump-points of the derivatives).

As to a peculiarity, the bumps in Fig. 7b correspond to sequences of neighboring vertices of the trend lines. Vertices were points of discontinuity (jump points) of the interaction coefficients, *g*_*i*_(*t*), of equation (1). In this case, the solutions of (1) were defined by successive systems of LV differential equations with constant coefficients, using the limits of *y*_*i*_ from the first system (with *t* approaching the jump point from below) as initial values for *y*_*i*_ in the second system. Therefore, starting in 2005, the market share followed a system of LV differential equations with constant interaction coefficients (the values *b* of Table 4) till the first vertex in 2013. At this moment of time (2013.24), the interaction coefficient for wind power jumped from −0.3664 (coefficient *b*) to −0.0655 (coefficient *d*); the other coefficients did not change. The LV dynamics continued with this new system of coefficients till the second point of discontinuity (2014.36), and so on. Notably, the vertices were concentrated around 2014 ± 0.8 and the trends of all products flattened to the right of their respective vertices. We checked if the LV model could be simplified by using a common vertex for all trends. However, this worsened the tests for “white noise”.

Summarizing, in Austria the market shares of green energy have been growing (Fig. 5). Distinguishing between traditional and emerging renewables (Fig. 6) identified the latter ones as drivers for this growth. Going into still more details (Fig. 7) we noted the growing importance of “other biofuels”, which together with hydropower became the main domestic energy products with a high potential for further growth of market shares. Further, extrapolating the model curves into the infinite future identified the high potential of photovoltaics (as for the latter model all interaction coefficients of the inside goods are negative).

#### 3.2.3 Electricity: worldwide

Next, we fitted LV models to the 1990 to 2019 data of [14] for the four groups of electricity produced from “fossils”, “nuclear”, “green”, and “remainder” (remainder of the other forms of electricity to the total electricity output), which we defined as the outside good. We fitted trigo-linear functions, equation (4e), to the utilities of these groups.

**Fig. 8:**
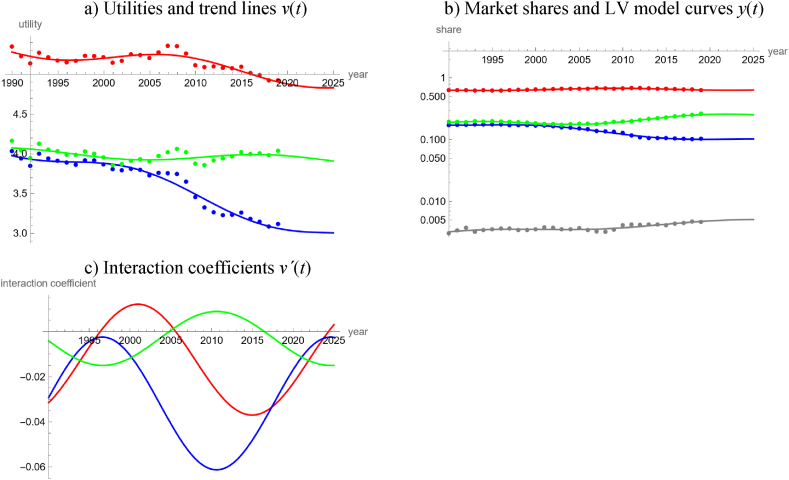
LV model for the global production of electricity from four groups of sources, in terms of utilities (8a), market shares (8b) and interaction coefficients (8c), based on data for 1990–2019 from Ref. [14] and model parameters in Table 6. Data (utilities and market shares) are displayed as dots and model curves as lines. Colors identify product groups: red (carbon-based fossils), blue (nuclear), green (renewables and waste) and gray (remainder as outside good). Figure b is a logarithmic plot. (For interpretation of the references to color in this figure legend, the reader is referred to the Web version of this article.)

As for the worldwide energy model of Table 1 and Fig. 4, we first optimized the period of each product. However, we ignored potential outliers (e.g., 1992 or 2008), as these were not evident from a visual inspection. For electricity from carbon-based fossils and nuclear electricity, the best-fit periods were close together (about 24.5 years), but for green electricity the utilities appeared to be linear. To construct a model like the energy model, we experimented with a common period for all electricity groups. We finally defined an arbitrary common period of 28 years; the so obtained model was more parsimonious (for all three time-series) than the model with optimized periods. It is summarized in Table 5 and Fig. 8a, b, and 8c. Except for nuclear, the R-squared values were rather small, because the utilities were close to a constant. However, the precision rates were excellent and the “white noise hypothesis” was satisfied. We therefore accepted the model.

**Table 5:** LV model for worldwide electricity production.

product group	$v\left(t\right)=a+b∙t+c∙\sin\left(\frac{2∙\pi }{28}∙t\right)+d∙\cos\left(\frac{2∙\pi }{28}∙t\right)$				White noise?		Good fit?	
	a	b	c	d	P_norm_	P_auto_	*R*^2^	PR
Fossils	30.1	−0.012	−0.11	−0.03	0.99	0.31	0.78	0.99
Nuclear	67.4	−0.032	−0.05	−0.12	0.97	0.17	0.96	0.98
Green	10.1	−0.003	0.02	0.05	0.89	0.24	0.36	0.98
Remainder	NA (outside good)							0.96

**Note:** Using the data [14] from 1990 to 2019, the table displays the best fit parameters (rounded to the last shown decimal) of trigo-linear trend lines and informs about the goodness of fit.

A common feature with the previous analysis for energy was the generally declining market share of electricity from nuclear fuels. However, it finally stabilized. While initially all product groups evolved in mutualism, in 1996 electricity from fossil sources became the sole predator and in 2005 green electricity became the sole predator. However, from 2016 onwards the interaction coefficients identified a longer period of mutualism that may lead in 2025 to a competitive edge for electricity from fossils. Thus, the market dynamics of this model did not suggest that globally 90 % green electricity could be achieved by 2050. The forecasts of this model for 2020 were 63.6 % for electricity from carbo-based fossils, 10.1 % for nuclear power, 25.8 % for green electricity, and 0.5 % for the remainder. The actual 2020 market shares were 61.6 %, 10.0 %, 28.0 %, and 0.4 %.

#### 3.2.4 Electricity: Austria

Referring to the [14] data, for Austria none of the five simple trends achieved an acceptable fit to either the 28-years or the 15-years series of logits of green electricity. We therefore expected a rather complicated market and devised a model for a short time span, ten years from 2011 to 2020, but with a detailed stratification for the sources used for electricity production, based on the data [55].

**Fig. 9:**
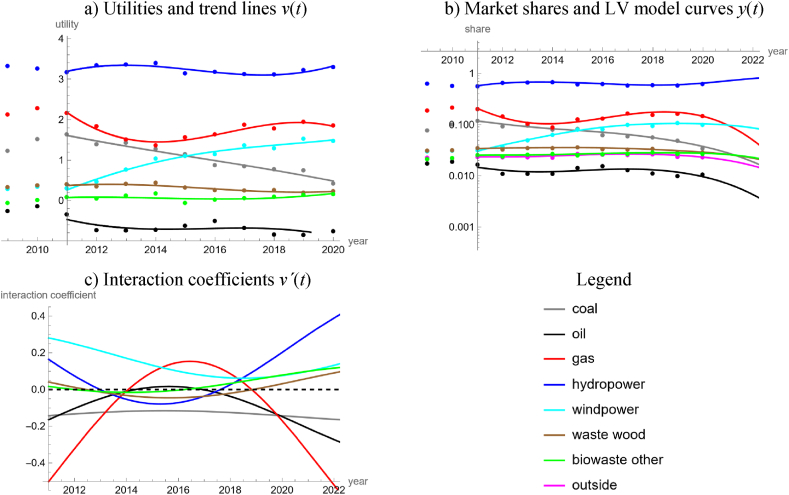
LV model for the Austrian production of electricity from various sources in terms of utilities (9a), market shares (9b) and interaction coefficients (9c), using data for 2011–2020 from Ref. [55] with model parameters of Table 6 (data as dots and models as lines).

Our model distinguished between electricity produced from coal (black, brown, and coal gas), oil, natural gas, hydropower, wind power, waste wood, and other biowaste (municipal waste, waste lye, landfill and sewage gas). In part, these raw materials were imported. The outside good collected the remainder (electricity from biogas and other biogenic fuels, photovoltaics, process heat and geothermal heat, industrial and municipal waste from nonrenewable sources). For the LV model, we fitted trigo-linear trends to the utilities, equation (4e), assuming a period of 20 years. Table 6 and Fig. 9a, b, 9c, summarize the outcomes. Thus, using 28 parameters, the model could provide a satisfactory description of nine time-series over ten years, each.

**Table 6:** LV model for electricity production in Austria.

Electricity from	$v\left(t\right)=a+b∙t+c∙\sin\left(\frac{2∙\pi }{20}∙t\right)+d∙\cos\left(\frac{2∙\pi }{20}∙t\right)$				White noise?		Good fit?	
	*a*	*b*	*c*	*d*	*P*_*norm*_	*P*_*auto*_	*R*^2^	*PR*
Coal	299.1	−0.15	0.10	0.01	0.60	0.11	0.96	0.94
Oil	378.8	−0.19	0.64	0.13	0.53	0.06	0.45	0.88
Gas	851.2	−0.42	1.64	0.80	0.56	0.54	0.92	0.93
Hydropower	−462.9	0.23	−0.98	−0.09	0.23	0.60	0.75	0.96
Wind power	−381.8	0.19	−0.18	−0.36	0.66	0.44	0.98	0.95
Waste wood	−102.0	0.05	−0.30	−0.06	0.99	0.17	0.83	0.96
Biowaste other	−116.9	0.06	−0.23	0.06	0.70	0.80	0.34	0.96
Outside good	NA (outside good)							0.96

**Note:** Using data for 2011–2020 from Ref. [55], the table displays the best fit parameters of trigo-linear trend lines and measures for the goodness of fit.

In 2011, all forms of green electricity (produced from hydropower, wind power, and wastes from biogenic sources) started with positive interaction coefficients and all forms of electricity from non-renewable sources (coal, gas, oil) with negative ones. However, soon green electricity lost its competitive edge, except for wind power, whose interaction coefficient remained positive all the time. From 2014 till the end of 2018, electricity from gas had positive interaction coefficients, followed till 2016 by oil, while the interaction coefficient for coal remained negative all the time. Finally, green electricity (hydropower, in particular) regained its competitive edge. By the end of 2018 all forms of green energy had positive interaction coefficients and the model forecasted this market dynamics till 2032: Thus, except for the outside good (a mixture of electricity from renewable and non-renewable sources), all forms of green electricity will compete, and they will predate on all forms of electricity from fossil sources, which in turn will evolve in mutualism. In view of this dynamics, 90 % green electricity could be easily achieved prior to 2050. This forecast was insofar plausible, as hydropower (currently 61 % of domestic electricity production) could be increased by 15 % till 2030 [72].

## 4 Discussion

Our study detected that with respect to the temporal evolution of the market shares of renewables the electricity market was more complex than the energy market. We arrived at this conclusion by a comparison of the percentages of countries, where neither the long-term or the short-term market share data of renewables could be described well by simple models (linear, quadratic, cubic, rational or trigonometric fits to the logits). For the electricity market, this rate was 33 % (15 of 45 countries, 95 % Clopper-Pearson [73] exact confidence limits 20–49 %) and for the energy market it was 8 % (3 of 40 countries, confidence limits 2–20 %). Counting datasets with no acceptable fit by a simple trend line, the respective rates were 50 % for electricity (45 of 90 datasets, confidence interval 39–61 %) and 41 % for energy (33 of 80 datasets, confidence limits 30–53 %), but the confidence intervals did overlap. A comparison of Fig. 4c for the worldwide energy market with Fig. 8c for the worldwide electricity market corroborate this observation. Similarly, in the case of Austria, a LV model with linear utilities fitted well to 20-years data about different energy products (Fig. 5), while for electricity a comparably complex model was needed to describe the evolution over merely ten years (Fig. 9).

We also collected evidence linking this difference in complexity to the ease of policy interventions.

1) The high accuracy of the worldwide energy model, Fig. 4, and its regular appearance suggest that the global energy market was immune to policy interventions. This is insofar plausible, as there may be too many actors with low coordination. We speculate that the regularity might come from a Juglar [74] business cycle, which in turn might be explained by investment decisions affecting production [75].
2) Further, for this model there were long phases of mutualism between all groups of energy products. While mutualism between renewable energy products may be desirable from a policy perspective, as it might reinforce their market penetration [53], the observed mutualism between renewable and non-renewable energy products (and the continuing dominance of carbon-based fossils) appears as incompatible with current pro-renewable policies.
3) LV models based on market shares in dollars identified an anomaly (structural break) related to the 2008 economic crisis [50,53]. For the energy models, Fig. 4, Fig. 5, Fig. 6, Fig. 7, we could not discern any anomaly around 2008. Perhaps, this was a consequence of the typical long-term contracts in the productive sector. This stability of the market despite an economic crisis was also plausible, as in general the production of energy-products cannot be easily switched on or off. (For example, flaring gas instead of selling it at lower prices might be neither economically sound nor environmentally sustainable.)
4) For the long-term model of energy production in Austria (Fig. 6) we could not discern any anomalies related to the market liberalization in 2001. Before 2001, consumers were assigned to regional monopolists that delivered electricity or gas at prices set by the government. In 2001 Austria opened the market for electricity and later for gas. This was reinforced by a series of laws issued between 2010 and 2013 (e.g., eco-electricity act, electricity act, energy control act, gas act, renewable energy expansion act) and the strengthening of the energy regulator (E-Control). Liberalization was a most massive policy intervention and producers were concerned about increased competition in the wider European market, allegedly leading to lower prices, less investments, and a reduction of domestic energy production. Perhaps the pricing mechanism did not generate sufficient incentives for the end-users to switch to renewable energy [76].

However, the following observations relativize this evidence.

1) As to item 1 above, before fitting the model we had removed two outliers, as the market shares for heat were comparably low in 1990 and 1991. We do not know the reason for its higher market shares since 1992 but we speculate that it may have been related to the fuel-switching policies of the 1990s [77]. In this case, past policies would have had an impact on the energy market, but our energy model of Fig. 4 ignored it and oversimplified (unless the policy did only have short-term effects).
2) Extrapolating the trends of the energy and electricity models of Fig. 2, Fig. 3, Fig. 4, Fig. 5 and 8 (modeling of international data [14]) to 2020 rather underestimated the actual market shares of renewables. We speculate that the increase in market shares for renewables (in comparison to the projections) could have been an indirect consequence of the worldwide Covid-policies ([78] for other impacts). This would indicate a susceptibility of the energy market for policy interventions. Further, there was no indication that the production of electricity was affected stronger (higher sensibility to policies) than that of energy.
3) As to item 3, for electricity we did not discern anomalies around 2008, either. However, we considered only one model (Fig. 8), where this anomaly could apply.
4) Similarly, concerning item 4, we did not observe an anomaly for the Austrian electricity market around 2001, but owing to the needed complexity we did not model it prior to 2011.
5) Around 2016, both the worldwide energy model (Fig. 4) and the worldwide electricity model (Fig. 8) displayed structural breaks. The interaction coefficient of fossil energy became positive, the interaction coefficient of fossil electricity started to increase, and the interaction coefficients of renewable energy and green electricity changed sign (though in opposite directions). Perhaps, this pattern reflected the price reductions for gas between 2015 and 2016. In this case, the energy market would appear to be as susceptible to external influences as the electricity market.
6) Energy studies of market shares in dollars identified an “unexplained structural break” around 2012–2013 [50,53]. Our models based on Austrian data [55] replicated this: Fig. 6c detects a break of the market dynamics in 2014 (fossils and traditional renewables changed from their long-lasting mutualism since 1980 to a predator-prey rivalry). Fig. 7a replicates this in a weaker form, as for all energy products there occurred a flattening of the trends for the utilities around 2014 ± 1 (clustering of vertices). Fig. 9 displays a clustering of breaks for the electricity market around 2014. However, using the data of [14], owing to different definitions of product groups we could not replicate these anomalies (Fig. 4, Fig. 5, Fig. 8). Thus, with respect to this potential anomaly there was no difference between the electricity and the energy market.

We conclude that the complexity of trends allows to discern between the markets for energy products and for electricity. However, more research is still needed to about our hypothesis that a higher complexity would be associated to an easier implementation of policies to control these markets. A difficulty for such research comes from the interpretation of the data. Different sources reported different time-series for the same market, although all data originated from a common source. Perhaps the institutions that collect and disseminate the data are not fully aware of these discrepancies. This leaves a knowledge gap and further research is needed to close it.

## Data availability statement and Supplementary Information

The data used for this paper are not stored in a repository. For, the international data are open access, available from Ref. [14], but the owners do only accept their publication in graphical form. The domestic data about Austria [55] are provided in a Supplementary Information in the form of the following Excel files:

• SupportingInformation_Energy.xlsx
• SupportingInformation_Electricity.xlsx

The outcomes of the fit of five simple trend lines to the logits of renewable energy and electricity data from 45 countries are summarized in Table S1 and Table S2 of a PDF File.

• SupportingInformation_LogitModels.pdf

Mathematica files for the computations are available on request from the first author.

## CRediT authorship contribution statement

**Norbert Brunner:** Conceptualization, Formal analysis, Investigation, Methodology, Software, Supervision, Validation, Visualization, Writing – original draft, Writing – review & editing. **Doris Straßegger:** Data curation, Investigation, Resources, Validation, Writing – original draft.

## Declaration of competing interest

The authors declare that they have no known competing financial interests or personal relationships that could have appeared to influence the work reported in this paper.

## References

[1] UN (2015). *Paris agreement*. Treaty collection, chapter XXVII, subchapter 7.d, united nations, New York, NY, USA, link. https://treaties.un.org/.

[2] Perissi I., Jones A. (2022). Investigating European union decarbonization strategies: evaluating the pathway to carbon neutrality by 2050. Sustainability.

[3] Wu X., Tian Z., Guo J. (2022). A review of the theoretical research and practical progress of carbon neutrality. Sustain. Operations and Comput..

[4] UNEP (2019).

[5] WMO (2022).

[6] IEA (2021).

[7] IPCC (2022).

[8] IRENA (2021). World energy transitions outlook: 1.5°C pathway, international renewable energy agency, abu dhabi. http://www.irena.org/publications.

[9] IEA (2021).

[10] An L., Zhao T.S., Li Y.S. (2015). Carbon-neutral sustainable energy technology: direct ethanol fuel cells. Renew. Sustain. Energy Rev..

[11] IEA (2021).

[12] Akaike H. (1974). A new look at the statistical model identification. IEEE Trans. Automat. Control.

[13] UNSD (2018).

[14] IEA (2023). http://www.iea.org/data-and-statistics/data-product/world-energy-statistics-and-balances.

[15] Lotka A.J. (1925).

[16] Volterra V. (1926). Variazioni e fluttuazioni del numero d’individui in specie animali conviventi. Memoria della Reale Accademia Nazionale dei Lincei.

[17] Kuşkaya S., Bilgili F. (2020). The wind energy-greenhouse gas nexus: the wavelet-partial wavelet coherence model approach. J. Clean. Prod..

[18] Ozcan B., Tzeremes P.G., Tzeremes N.G. (2020). Energy consumption, economic growth and environmental degradation in OECD countries. Econ. Modell..

[19] Pouillet C. (1838). Mémoire sur la chaleur solaire, sur les pouvoirs rayonnants et absorbants de l'air atmosphérique, et sur la température de l'espace. Compte Rendu des Séances de l'Académic des Sciences.

[20] Van der Veen C.J. (2000). Fourier and the “greenhouse effect”. Polar Geogr..

[21] Zhang Q., Raza Shah S.A., Yang L. (2022). Modeling the effect of disaggregated renewable energies on ecological footprint in E5 economies: do economic growth and R&D matter?. Appl. Energy.

[22] Hubbert M.K. (1959). 44th Annual Meeting of the American Association of Petroleum Geologists.

[23] Mar-Molinero C. (1980). Tractors in Spain: a logistic analysis. J. Oper. Res. Soc..

[24] Brandt A.R. (2007). Testing Hubbert. Energy Pol..

[25] IEA (2022).

[26] IEA (2022). Press release about the ministerial meeting of 24.03.2022. http://www.iea.org/news/at-iea-ministerial-meeting-global-energy-leaders-vow-to-strengthen-energy-security-and-accelerate-clean-energy-transitions.

[27] Narayan S., Doytch N. (2017). An investigation of renewable and non-renewable energy consumption and economic growth nexus using industrial and residential energy consumption. Energy Econ..

[28] Agresti A. (2013).

[29] Bass F.M. (1969). A new product growth for model consumer durables. Manag. Sci..

[30] Abrams P.A. (2000). The evolution of predator-prey interactions: theory and evidence. Annu. Rev. Ecol. Systemat..

[31] Murray J.D. (1993).

[32] Goodwin R.M., Feinstein C.H. (1967). Socialism, Capitalism and Economic Growth. Essays Presented to Maurice Dobb.

[33] Kim J., Lee D.J., Ahn J. (2006). A dynamic competition analysis on the Korean mobile phone market using competitive diffusion model. Comput. Ind. Eng..

[34] Michalakelis C., Sphicopoulos T.S., Varoutas D. (2011). Modelling competition in the telecommunications market based on the concepts of population biology. Transactions on Systems, Man and Cybernetics. Part C: Appl. Rev..

[35] Modis T. (1999). Technological forecasting at the stock market. Technol. Forecast. Soc. Change.

[36] Dang H.S., Huang Y.F., Wang C.N., Nguyen T.M.T. (2016). An application of the short-term forecasting with limited data in the healthcare traveling industry. Sustainability.

[37] Fu X., Zhang P., Zhang J. (2017). Forecasting and analyzing internet users of China with lotka-volterra model. Asia Pac. J. Oper. Res..

[38] Tsai B.H., Chang C.J., Chang C.H. (2016). Elucidating the consumption and CO2 emissions of fossil fuels and low-carbon energy in the United States using Lotka-Volterra models. Energy.

[39] Fthenakis V.M., Kim H.C. (2007). Greenhouse-gas emissions from solar electric- and nuclear power: a life-cycle study. Energy Pol..

[40] Marasco A., Picucci A., Romano A. (2016). Market share dynamics using lotka-volterra models. Technol. Forecast. Soc. Change.

[41] Romano A. (2016). A study of tourism dynamics in three Italian regions using a nonautonomous integrable lotka-volterra model. PLoS One.

[42] Marasco A., Romano A. (2018). Inter-port interactions in the le havre-hamburg range: a scenario analysis using a nonautonomous Lotka Volterra model. J. Transport Geogr..

[43] Marasco A., Romano A. (2018). Deterministic modeling in scenario forecasting: estimating the effects of two public policies on intergenerational conflict. Qual. Quantity: Int.J. Methodol..

[44] Horgan D.C. (2020). Modelling market share in the UK grocery retail sector using the nonautonomous Lotka-Volterra equations. http://www.researchgate.net.

[45] Focacci C.N., Quintavalla A. (2020). Unpredictable spillovers among water uses? An analysis of agricultural, industrial, and household uses of water in the Balkans. PLoS One.

[46] Sotis C. (2021). How do google searches for symptoms, news and unemployment interact during COVID-19? A lotka-volterra analysis of google trends data. Qual. Quantity: Int.J. Methodol..

[47] Ziegler A.M., Brunner N., Kühleitner M. (2020). The markets of green cars of three countries: analysis using lotka-volterra and bertalanffy-pütter models. J. Open Innovation: Technol., Market and Complexity.

[48] Bauer R., Schwarzmayr F., Brunner N., Kühleitner M. (2022). Dynamics of the Austrian food market: application of lotka-volterra differential equations. Open J. Model. Simulat..

[49] Brunner N., Das S., Starkl M. (2023). Lotka-Volterra analysis of river Ganga pollution in India. Ecol. Indicat..

[50] Bondia R., Ghosh S., Kanjilal K. (2016). International crude oil prices and the stock prices of clean energy and technology companies: evidence from non-linear cointegration tests with unknown structural breaks. Energy.

[51] Managi S., Okimoto T. (2013). Does the price of oil interact with clean energy prices in the stock market?. Jpn. World Econ..

[52] Reboredo J.C., Rivera-Castro M.A., Ugolini A. (2017). Wavelet-based test of co-movement and causality between oil and renewable energy stock prices. Energy Econ..

[53] Dominioni G., Romano A., Sotis C. (2019). A quantitative study of the interactions between oil price and renewable energy sources stock prices. Energies.

[54] Focacci C.N. (2021). Technological unemployment, robotisation, and green deal: a story of unstable spillovers in China and South Korea (2008–2018). Technol. Soc..

[55] Austria Statistics (2022). https://statcube.at/statistik.at/ext/statcube.

[56] Pacesila M., Burcea S.G., Colesca S.E. (2016). Analysis of renewable energies in European Union. Renew. Sustain. Energy Rev..

[57] National-Park (2017). Nationalpark donau-auen. Österreichische Wasser- Abfallwirtsch..

[58] Sani A.K., Singh R.M., Amis T., Cavarretta I. (2019). A review on the performance of geothermal energy pile foundation, its design process and applications. Renew. Sustain. Energy Rev..

[59] Zupančič M., Možic V., Može M., Cimerman F., Golobič I. (2022). Current status and review of waste-to-biogas conversion for selected European countries and worldwide. Sustainability.

[60] FM:ST (2019). Integrated national energy and climate plan for Austria. Government of Austria, federal Ministry of sustainability and tourism, Vienna, Austria. https://ec.europa.eu/energy/sites/ener/files/documents/at_final_necp_main_en.pdf.

[61] Van de Graaf T., Haesebrouck T., Debaere P. (2018). Fractured politics? The comparative regulation of shale gas in Europe. J. Eur. Publ. Pol..

[62] Romano A. (2013). Turning the coin: a competition model to evaluate mergers. J. Res. Industrial Organization.

[63] Kloppers P.H., Greeff J.C. (2013). Lotka-Volterra model parameter estimation using experiential data. Appl. Math. Comput..

[64] Marasco A., Picucci A., Romano A. (2016). Determining firms' utility functions and competitive roles from data on market shares using lotka-volterra models. Data Brief.

[65] Wolfram Research (2023). Mathematica, version 13.3. Wolfram research inc., champaign. http://www.wolfram.com/mathematica.

[66] Potra F.A., Wright S.J. (2000). Interior-point methods. J. Comput. Appl. Math..

[67] Spiess A.N., Neumeyer N. (2010). An evaluation of R^2 as an inadequate measure for nonlinear models in pharmacological and biochemical research: a Monte Carlo approach. BMC Pharmacol..

[68] Burnham K.P., Anderson D.R. (2002).

[69] Diebold F.X. (2007).

[70] Box G.E.P., Pierce D.A. (1970). Distribution of residual autocorrelations in autoregressive-integrated moving average time series models. J. Am. Stat. Assoc..

[71] Newey W.K., West K.D. (1987). A simple, positive semi-definite, heteroskedasticity and autocorrelation consistent covariance matrix. Econometrica.

[72] IHA (2022). http://www.hydropower.org/country-profiles/austria.

[73] Clopper C.J., Pearson E.S. (1934). The use of confidence or fiducial limits illustrated in the case of the binomial. Biometrika.

[74] Juglar C. (1862).

[75] Iwamoto M., Iwamoto C., Yamada T. (1999).

[76] Dolna-Gruber C., Knaus K., Zwieb L. (2022). http://www.energyagency.at.

[77] Pettersson F., Söderholm P., Lundmark R. (2012). Fuel switching and climate and energy policies in the European power generation sector: a generalized Leontief model. Energy Econ..

[78] Balabanyan A., Semikolenova Y., Hankinson D., Nash S., Parcels C. (2021). https://openknowledge.worldbank.org/handle/10986/36179.

