# Supplementary material for: Lotka-Volterra models for the market penetration of renewable energy
Source: Heliyon. 2023 Nov 23;9(12):e22704. doi: 10.1016/j.heliyon.2023.e22704 (PMC10711125; doi:10.1016/j.heliyon.2023.e22704)
Supplement: Multimedia component 3 [file mmc3.pdf]

Appendix: Outcomes of fitting simple trends to the logits

Table S1: When will 90% of domestic energy production be renewable?

Country	Data (years)	Linear	Quadratic	Cubic	Rational	Trigo-nometric	Country	Data (years)	Linear	Quadratic	Cubic	Rational	Trigo-nometric
Australia	15	NA[a]	NA	NA	NA	NA	Ireland	15	NA	NA	NA	NA	NA
	30	NA	NA	NA	NA	>[b, c]		30	NA	NA	>[c]	NA	>[b]
Austria	15	2024	2021	2021	2021[b]	2021	Israel	15	NA	NA	2024	NA	>b
	30	2027	2022	2021	2021[b]	2021		30	NA	NA	2027	NA	2033[b]
Belgium	15	2047	NA	>	>	>[b]	Italy	15	2031	NA	2025	>[b]	>
	30	NA	2035	>	2033	>[b]		30	NA	NA	>	NA	>[b]
Brazil	15	>	>	2030[b]	>	>	Japan	15	NA	NA	>	NA	>[b]
	30	NA	NA	NA	NA	NA		30	NA	NA	NA	NA	NA
Canada	15	NA	NA	>[b]	NA	>	Korea	15	>[b]	NA	>	>	>
	30	>	>	>[b]	>	>		30	NA	2037[b]	2040	2026	>
Chile	15	NA	NA	NA	NA	NA	Lithuania	15	NA	NA	>	2020	>[b]
	30	2030	>	NA	>[b]	>		30	NA	NA	NA	NA	NA
China	15	NA	NA	>	NA	>[b]	Mexico	15	NA	NA	2043	2026	>[b]
	30	NA	NA	NA	NA	NA		30	NA	NA	2035[b]	NA	NA
Colombia	15	NA	NA	NA	NA	>[b]	Netherlands	15	2050	NA	2029[b]	2024	2030
	30	NA	NA	NA	NA	NA		30	>	NA	2029[b]	NA	NA
Czech Republic	15	>	NA	>	>	>[b]	New Zealand	15	>	2034	>[b]	2028	>
	30	>	>	2042	2045[b]	>		30	NA	2039[b]	2048	2026	>
Denmark	15	2043[b]	NA	2039	2039	2050	Norway	15	NA	NA	NA	NA	NA
	30	NA	NA	2037	NA	>[b]		30	NA	NA	>[b]	NA	NA
Estonia	15	>	NA	2031	>[b]	2035	Poland	15	>	NA	2038	>[b]	>
	30	NA	NA	2026[b]	>	NA		30	>[b]	>	2038	2053	>
Finland	15	NA	NA	NA	NA	NA	Slovak Republic	15	>	NA	2028[b]	>	>
	30	>[b]	>	2036	2043	>		30	NA	NA	NA	NA	NA
France	15	>	NA	2041	>[b]	>	Slovenia	15	NA	NA	NA	NA	NA
	30	NA	2048	>	NA	>[b]		30	>	>	>	>	>[b]
Germany	15	2040	NA	2028[b]	>	>	South Africa	15	NA	NA	NA	NA	NA
	30	NA	NA	NA	NA	NA		30	NA	>	2043	>	>[b]
Greece	15	2044	NA	>[b]	2026	>	Spain	15	2042	NA	>	>	>[b]
	30	NA	2033	2027[b]	2023	2033		30	2046	2034	>	2033	>[b]
Hungary	15	>	NA	>	>	>[b]	Thailand	15	NA	NA	2036[b]	2026	2045
	30	NA	NA	>	NA	>[b]		30	NA	NA	>[b]	NA	NA
India	15	NA	NA	>	NA	>[b]	Turkiye	15	NA	NA	>[b]	NA	>
	30	>	>	2043[b]	>	>		30	NA	NA	NA	NA	NA
Indonesia	15	>	NA	2035	>	>[b]	UK	15	2042	NA	>	>	>[b]
	30	NA	NA	NA	NA	NA		30	NA	NA	>	NA	>[b]
USA[d]	15	NA	NA	>[b]	NA	>	USA	30	NA	NA	NA	NA	NA

Note: Using the data IEA (2023) as described in the text, the table displays the year, when renewables will reach 90% market share (decimals omitted) according to the stated model; [a]) NA indicates that the model was not accepted (countries with 100% renewable energy production or with 10 "NA" were not listed); [b]) this was the outcome of the most parsimonious model (amongst the five models considered for these data), [c]) >indicates that market shares of 90% will not be reached prior to 2050; [d]) results for the USA in a line.

Table S2: When will 90% of produced electricity be renewable?

Country	Data (years)	Linear	Quadratic	Cubic	Rational	Trigo-nometric	Country	Data (years)	Linear	Quadratic	Cubic	Rational	Trigo-nometric
Australia	15	>[c]	NA[a]	>[b]	2028	>	Italy	15	2041	NA	>	>	>[b]
	28	NA	2038	2034[b]	NA	2038		28	NA	NA	NA	NA	NA
Belgium	15	2036	NA	>	>	>[b]	Japan	15	>	NA	>	2025	>[b]
	28	NA	NA	>	NA	>[b]		28	NA	2044	2032[b]	NA	2044
Brazil	15	NA	NA	NA	NA	NA	Korea	15	>	NA	2031[b]	2024	2032
	28	NA	2039[b]	2047	>	2039		28	NA	NA	2029[b]	NA	2037
Canada	15	>[b]	>	2040	>	>	Lithuania	15	NA	NA	>[b]	NA	>
	28	NA	2043	2047	NA	>[b]		28	NA	NA	NA	NA	NA
Chile	15	NA	NA	NA	NA	NA	Luxem-bourg	15	NA	NA	2020	2019	2020[b]
	28	NA	NA	>	>[b]	NA		28	NA	NA	NA	NA	NA
China	15	>	NA	>	2039	>[b]	Nether-lands	15	>	NA	2029[b]	2033	2049
	28	NA	2043	2034[b]	NA	2043		28	NA	>[b]	>	>	>
Czech Republic	15	>	NA	>	>	>[b]	New Zealand	15	2025[b]	NA	>	2027	>
	28	NA	2040	>	NA	>[b]		28	NA	NA	NA	NA	NA
Denmark	15	2025	NA	>[b]	2022	>	Norway	15	NA	NA	NA	NA	NA
	28	2026[b]	NA	2022	2024	2025		28	NA	NA	>>[d]	NA	>>[b]
Finland	15	NA	NA	>[b]	2032	>	Poland	15	2043	NA	>	>	>[b]
	28	NA	NA	NA	NA	NA		28	NA	NA	NA	NA	NA
France	15	>[b]	NA	2035	>	>	Singapore	15	>	NA	>	2031	>[b]
	28	NA	NA	NA	NA	NA		28	NA	NA	NA	NA	NA
Germany	15	2040[b]	NA	2031	2045	2050	Slovak Republic	15	NA	NA	>	>[b]	>
	28	2043	2033	>[b]	2032	>		28	NA	NA	NA	NA	NA
Greece	15	2043[b]	NA	>	2043	>	Spain	15	NA	NA	>	>	>[b]
	28	NA	NA	2031	2026[b]	2035		28	NA	NA	NA	NA	NA
Hungary	15	>[b]	NA	2045	>	>	Thailand	15	NA	NA	2026	2022[b]	2031
	28	NA	NA	NA	NA	NA		28	NA	NA	2028[b]	NA	NA
Iceland	15	NA	NA	NA	NA	NA	UK	15	2032	NA	>	2027	>[b]
	28	>>[d]	>>	2036	>>	>>[b]		28	NA	2026[b]	2027	2023	2028
Ireland	15	>	NA	2029	>[b]	>	USA	15	>[b]	NA	>	2044	>
	28	NA	2029	>	NA	>[b]		28	NA	2040[b]	2039	NA	>

Note: Using the data IEA (2023) as described in the text, the table displays the year, when renewables will reach 90% market share (decimals omitted) according to the stated model; [a]) NA indicates that the model was not accepted (countries with 10 "NA" were not listed); [b]) this was the outcome of the most parsimonious model (amongst the five models considered for these data), [c]) >indicates that market shares of 90% will not be reached prior to 2050; [d]) >> indicates that till 2050 the market share will remain above 90%.
